# Primary surgery versus chemoradiotherapy for advanced oropharyngeal cancers: a longitudinal population study

**DOI:** 10.1186/1916-0216-42-31

**Published:** 2013-04-22

**Authors:** Daniel O’Connell, Hadi Seikaly, Russell Murphy, Charles Fung, Tim Cooper, Aaron Knox, Rufus Scrimger, Jeffrey R Harris

**Affiliations:** 1 Division of Otolaryngology-Head & Neck Surgery, University of Alberta, Edmonton, AB, Canada; 2 Faculty of Medicine and Dentistry, University of Alberta, Edmonton, AB, Canada; 3 Division of Radiation Oncology, University of Alberta, Edmonton, AB, Canada; 4 1E4.29 Walter C. MacKenzie Health Sciences Centre, 8440 112 Street, Edmonton, AB T6G 1B7, Canada

## Abstract

**Background:**

Treatment for advanced stage oropharyngeal squamous cell carcinoma (OPSCC) includes combined chemoradiation therapy or surgery followed by radiation therapy alone or in combination with chemotherapy. The goal of this study was to utilize available evidence to examine survival outcome differences in patients with advanced stage OPSCC treated with these different modalities.

**Methods:**

Patients with advanced stage OPSCC were identified. Primary outcome measurements were disease specific and overall survival rates with differences examined via Kaplan-Meier and logistic regression analysis.

**Results:**

344 patients were enrolled. 94 patients underwent triple modality therapy inclusive of surgery followed by adjuvant combined chemotherapy and radiation therapy (S-CRT). 131 had surgery and radiation therapy (S-RT), while 56 had chemoradiation (CRT) therapy as their primary treatment. A total of 63 patients had single modality radiation therapy and were excluded from analysis due to the large number of palliative patients.

Kaplan-Meier overall survival analysis showed that therapy with S-CRT had the highest disease specific survival at five years (71.1%). This is contrasted against S-RT and CRT, with five year survival rates at 53.9%, and 48.6%, respectively.

Cox regression showed that the comparison of S-CRT vs. S-RT, and CRT is associated with statistically significant increased hazard ratios of 1.974, and 2.785, indicating that both S-RT and CRT are associated with a reduced likelihood of survival at 5 years when compared to S-CRT.

**Conclusions:**

In this population based cohort study S-CRT is associated with a 17–22% 5 year disease specific survival benefit compared to CRT or S-RT.

## Introduction

Oropharyngeal Squamous Cell Carcinoma (OPSCC) is defined as epithelial cell derived cancers occurring within the confines of the soft palate superiorly to the hyoid bone inferiorly. Structures included in this area are the base of tongue, tonsillar pillars, pharyngeal walls, and soft palate. Any cancer treatment process affecting this area often has negative implications for the patients’ swallowing, speech, and breathing functions. Current literature shows OPSCC being associated with a poor survival prognosis. Several factors are suggested cause this high level of mortality. The inaccessibility of this site causes most tumors to remain asymptomatic until they grow large enough to cause significant loss of function, often with nodal or distant metastasis [1].

Prior to advances in transoral resection techniques as well as microvascular reconstruction surgical extirpation of tumors of involving the oropharynx often resulted in large cosmetic and functional defects. In order to minimize possible morbidity, many centers moved away from primary surgery and towards combined CRT techniques in an attempt to avoid surgical techniques which are believed to cosmetically and functionally unacceptable. However, evidence now exists showing that primary surgery for OPSCC with microvascular free flap reconstruction can preserve function while maintaining excellent survival rates [2-5].

Large scale retrospective studies examining survival outcomes stratifying treatment modalities in one subsite of OPSCC revealed a statistically significant survival benefit associated with surgery being used as a treatment modality (alone or combined with RT) compared to RT alone or combined CRT [1]. A single study attempted to provide level 1 evidence to elucidate any significant differences in survival in patients treated with surgery and adjuvant radiotherapy (S-RT) compared to concurrent CRT in stage III and IV non-metastatic SCC [6]. Unfortunately the study was terminated prior to meeting sufficient power due to slow patient accrual. Analysis of existing data revealed no significant benefit of combined CRT compared to S-RT in terms of long-term disease specific survival. Two large randomized studies examining post-operative RT versus combined CRT revealed a significant long-term survival benefit with combined CRT [7,8].

A systematic review examining the best evidence of surgical treatments of OPSCC in the current literature showed an improved survival in patients treatment with multimodality treatment comprised of surgical resection followed by combined chemotherapy and radiotherapy (S-CT/RT) compared to S alone or S-RT [9].

Confusing the issue of optimum treatment(s) for advanced OPSCC is the role of the Human Papilloma Virus (HPV) in oncogenicity of OPSCC. Landmark studies have shown that HPV positivity is associated with increased rates of OPSCC in patients with no other risk factors for head and neck cancer [10] as well as having improved survival compared to HPV negative OPSCC [11]. There are many debates over whether or not treatment strategies should be altered based on HPV status, although no strong evidence currently supports the safety or efficacy of doing this [12]. Furthermore as no standardized treatment for advanced OPSCC is currently accepted it poses a challenge to advocate for changing practice based on HPV status.

Current NCCN guidelines recommend CRT as the treatment of choice for advanced stage OPSCC with S-RT and S-CRT also being listed as acceptable treatment options [13-28]. No level I evidence exists in the literature that favours CRT over S-RT or S-CRT as treatment modalities of choice in OPSCC, rather the widespread usage of CRT in OPSCC stems from extrapolations of trials examining the efficacy of organ preserving treatment protocols using different combinations of chemotherapy and radiation fractionation patterns including the VA trial and RTOG 9111 [29,30]. Different cancer treatment centers continue to use different combinations of treatment modalities to treat these types of cancers. NCCN guidelines have recommended CRT as the first line treatment in advanced OPSCC in the absence of any comparative trials. As level I evidence comparing drastically different treatment paradigms will likely never be generated from a feasibility standpoint it is now imperative to use the best available evidence to examine survival outcomes of different treatment modalities. We now need to reflect upon the changing management paradigm to see if we have improved the outcome for this difficult to treat patient population.

The current study represents a systematic analysis of a prospectively collected population based database encompassing all of the OPSCC diagnosed between January 1, 1998 and December 31, 2009 in a single territorial region (northern Alberta) of Canada. All patients diagnosed with OPSCC within the province of Alberta are treated at one of two tertiatry care facilities. This cohort represents all patients treated at one of those two centres. The data represents current patterns of patient presentation, demographics, treatment strategies, and survival outcomes. The goal of the current analysis is to examine which treatment strategies provide the optimal survival outcome for patients diagnosed with OPSCC within the specified region with the hope of further improving cancer care.

## Materials and methods

Ethics approval was received prior to initiating this study from the University of Alberta Health Ethics Research Office, study # Pro 00001800, legacy #6843. The Alberta Cancer Registry (ACR) maintains certification through the North American Association for Central Cancer Registries (NAACCR). The ACR records and tracks all new diagnoses of cancer, their treatments, and deaths within the province of Alberta, Canada. All data is collected by the ACR through mandatory reporting of cancer by surgical pathology laboratories, enforced by provincial legislation. The methodology data collection and database maintenance is well documented [31,32].

All patients diagnosed with OPSCC and treated with their definitive therapy in Edmonton, Alberta between January 1^st^, 1998 and December 31^st^, 2009 were included in the analysis. Advanced OPSCC was defined as those with stage III and IV disease. Extraction codes for cancers involving the oropharynx, base of tongue, palate, tonsil, and “oral, pharynx, unspecified” were utilized. These files were then reviewed manually, both in electronic and paper forms. Data points pertaining to patient demographics, location of treatment, and treatment methods were collected. Clinical staging was done according to the American Joint Committee on Cancer (AJCC) staging system for cancer of the oropharynx. Treatment modalities used included radiotherapy (RT), concomitant chemotherapy and radiotherapy (CRT), surgery with adjuvant radiotherapy (S-RT), and surgery with adjuvant chemotherapy and radiation (S-CRT). Surgery involved both primary site ablation with locoregional or free tissue transfer reconstruction and unilateral or bilateral neck dissections. Neck dissection alone was not included in the surgical group. Chemotherapy was defined as patients receiving any single or combined agent therapy at any point in relation to surgery and/or radiation. Radiotherapy included all patients receiving fractionated, hyper-fractionated, or intensity modulated ration therapy.

Survival analysis involved dividing patients into their treatment groups: either S-RT, S-CRT, or CRT. All treatment modality groups were based on intent to treat protocols. Patients that were enrolled in a CRT treatment strategy who then required salvage surgery were included in the CRT group for the purposes of survival analysis. Longitudinal data from the Alberta Cancer Registry was exploited making use of the data of diagnosis as the starting point for survival time. Both Disease Specific Survival (DSS) and Overall Survival (OS) were extracted and confirmed from the database. Disease Specific Survival (DSS) was defined as all deaths directly attributed to OPSCC causes and complications. Overall Survival (OS) was defined as death from any and all causes. Tests used include the following: the Kruskall-Wallis test, in order to examine differences in age and gender between treatment groups; the Wilcoxon and log rank statistic to determine statistically significant associations with survival rates as stratified by age and gender, and the Cox regression multivariate analysis to determine significant independent association of treatment groups vs. survival outcomes.

## Results

A total of 344 patients with advanced stage OPSCC were enrolled sequentially through the multidisciplinary head and neck treatment clinic at the Cross Cancer Institute between January 1^st^, 1998 and December 31^st^, 2009.

### Patient demographics

Patient demographics for 344 patients with advanced stage OPSCC over an 11 year span are presented in Table 1. 94 patients were treated with S-CRT, with males representing 85.1% and females 14.9%. Average age in this all modality group was 54.59+/− 8.48 years. The S-RT group had 131 patients, made up of 77.9% men and 28% women. Average age was 56.77+/−10.30. the CRT group had 56 patients, with 78.6% being male and 21.4% female, and an average age of 58.5+/− 10.43. RT alone consisted of 71.7% men and 31.7% women. Average age in the RT group was 69.11+/11.41. Please note that the RT group was excluded from survival analysis as a significant number were treated with palliative intent. The Kruskal-Wallis test showed no statistical differences between the S-CRT, S-RT and CRT groups in regards to gender. The age distribution was found to be significantly different ((H)2 = 65.15, p < 0.001).

**Table 1:** Patient demographic and staging data advanced OPSCC

**Treatment**	**S-CRT**	**S-RT**	**CRT**	**RT**	**Total**
N	94	131	56	63	344
Sex-no (%)					
Male	80	29	12	20	75
Female	14	102	44	43	269
Age-years					
Mean+/− SD	54.69+/−8.48	56.77+/−10.30	58.5+/−10.43	69.11+/−10.41	58.74+/− 11.07

N – number of patients.

RT – radiotherapy.

CRT- combined chemotherapy and radiation therapy.

S-RT – surgical resection +/− reconstruction followed by adjuvant radiotherapy.

S-CRT – surgical resection +/− reconstruction followed by combined adjuvant chemotherapy and radiotherapy.

### Treatment characteristics

All patients undergoing surgery for a diagnosis of OPSCC all had resections of the primary tumor site with reconstruction via secondary intention, locoregional and/or free tissue reconstruction. Patients included in the S-CRT and S-RT arms of this study all had surgical resections of their primary site, with or without neck dissections followed by adjuvant radiation therapy with or without concomitant chemotherapy. Data on type of surgery and/or reconstruction is not contained within the ACR and is outside the scope of this particular study. Patients undergoing radiotherapy as part of their OPSCC treatment had varying protocols of fractionated, hyper-fractionated, and IMRT type external beam radiation. Current practice at our treatment center is 6600–7000 Gy dosing in CRT protocols, with 6000 Gy +/− 600 Gy boost dosing to the primary site in post-operative (S-RT, S-CRT) treatment protocols. Once again complete dosing, fractionation and delivery mechanism of radiation for each patient is outside the scope of this study. Patients undergoing chemotherapy as a component of treatment had varying combinations of platinum based chemotherapy agents, 5-fluorouracil, doxorubicin, and/or taxanes. Information where available revealed the majority of patients were treated with cisplatin or carboplatin based protocols. Complete data on treatment type and successful completion of dosing was outside the scope of the study.

### Survival outcomes

Overall and disease specific survival data at two and five years with stratification into treatment modalities for advanced stage OPSCC is found in Table 2. Log rank and Wilcoxon rank sum tests show a statistically significant association between survival time and treatment modality used to treat the disease (p < 0.001). S-CRT is found to have a two year disease specific survival of 90.1% and a five year of 71.1%. S-RT, and CRT are associated with two and five year disease specific survivals of 73.7% and 53.9%, 57.4% and 48.6% respectively.

**Table 2:** Kaplan-Meier disease specific and overall survival advanced (stage III/IV) OPSCC stratified based on treatment modality

**Stage**	**Treatment**	**Disease specific survival (%)**		**Overall survival (%)**	
		**2 year**	**5 year**	**2 year**	**5 year**
III/IV	S-CRT	90.1	71.1	87.7	63.1
III/IV	S-RT	73.7	53.9	69.7	47.4
III/IV	CRT	57.4	48.6	51.7	39.8
III/IV	RT	39.3	23.2	33.3	21.1

OPSCC – oropharyngeal squamous cell carcinoma.

RT – radiotherapy.

CRT- combined chemotherapy and radiation therapy.

S-RT – surgical resection +/− reconstruction followed by adjuvant radiotherapy.

S-CRT – surgical resection +/− reconstruction followed by combined adjuvant chemotherapy and radiotherapy.

Cox regression analysis was used to compare overall survival with the three treatment strategies. This revealed a significant association between disease specific survival and the treatment modality used (p < 0.0.0001). Comparison between the S-CRT group and all other modalities are demonstrated in Table 3. This data shows that, when compared to S-CRT, the hazard ratios are: 1.97 (p = 0.011) for S-RT; and 2.79 (p = 0.001) for CRT indicating an increased likelihood of succumbing to disease when treated with S-RT and CRT compared to S-CRT and is shown figuratively in graph 1.

**Table 3:** Cox-regression analysis of survival difference in stage III/IV OPSCC stratified based on treatment strategy, stage of cancer included as covariate

			**95% confidence interval**	
**Treatment**	**HR**	**p value**	**Lower**	**Upper**
S-CRT	baseline	baseline	baseline	baseline
S-RT	1.974	0.011	1.170	3.330
CRT	2.785	0.001	1.525	5.086
RT	5.398	<0.0001	3.128	9.315

OPSCC – oropharyngeal squamous cell carcinoma.

RT – radiotherapy.

CRT- combined chemotherapy and radiation therapy.

S-RT – surgical resection +/− reconstruction followed by adjuvant radiotherapy.

S-CRT – surgical resection +/− reconstruction followed by combined adjuvant chemotherapy and radiotherapy.

HR – hazard ratio.

## Discussion

OPSCC has long been associated with a poor prognosis as it often presents in an advanced stage. Due to its rarity, longitudinal population based prospective databases like the ACR remain one of the best tools for examining survival outcomes of contemporary cases of OPSCC treated within a territorial region. Although cancer registries such as the ACR involve prospective collection of longitudinal data, this retrospective analysis requires caution in interpretation due to potential confounding factors such as selection bias of treatment based on treatment location, treatment group stratification based on intent-to-treat protocols rather than completed treatment protocols, as well as the paucity of some information including pre-treatment performance status and co-morbidities [1]. However population based studies such as the one presented herein, do minimize the previous mentioned biases by including all patients in a contained population with treatment protocols considered within the standard of care, and do represent the best surrogate currently available for randomized trials that are not practical on certain populations.

The vast majority of oropharyngeal carcinomas identified in the ACR were SCC (>96%). Similar to previous published studies the majority of the cases were diagnosed in advanced stages 83% of patients were stage III or IV at time of diagnosis [1,9,13,16].

Two separate studies examining treatment outcomes in base of tongue SCC revealed 71% of patients presented with stage IV disease while up 81% of patients presented with stage III or IV disease [33,34]. A meta-analysis comparing S-RT vs. RT in the treatment of orophrayngeal cancers showed in most studies stage IV disease was the most common stage of presentation [14].

Optimal treatment of OPSCC remains controversial. Different cancer treatment centers worldwide advocate for surgery, radiotherapy, and chemotherapy alone or in different combinations [3-28,33-36]. A publication based on large scale phase III trials in advanced OPSCC comparing RT to CRT revealed 5 year disease specific survivals of 27% compared to 22%. A grouped analysis of single Phase III trials examining the efficacy of post-operative concomitant chemotherapy and radiotherapy compared to post-operative radiotherapy revealed 5 year disease specific survival of < 50% in both S-RT and S-CT/RT [7]. Analysis of the European Organization for Research and Treatment of Cancer (EORTC) randomized trial examining survival in advanced stage OPSCC treated with post-operative RT compared to post-operative CRT revealed progression free 5 year survival of 47% in the S-CRT arm compared to 36% in the S-RT arm of the study [8].

Although comparing the findings of these contemporary studies to the results reported here must be done with caution due to inherent differences in results of randomized trials and analysis of cancer databases with regards to disease specific survival, stark contrasts can be noted. Advanced stage OPSCC treated in Alberta with S-CRT can expect a predicted 2 year disease specific survival of 90.1% and a predicted 5 year disease specific survival of 71.1%. Patients treated with dual modality therapy either CRT or S-RT can expect 2 year disease specific survivals of 57.4% and 73.7% with 5 year disease specific survivals of 48.6% and 53.9% respectively (see Table 2). Pairwise comparisons examining survival outcomes of patients treated with CRT and S-RT compared to S-CRT reveal statistically significant hazard ratios of 2.79 (p = 0.001) and 1.97 (p = 0.015). This indicates an almost three fold increased risk of succumbing to OPSCC when treated with CRT compared to S-CRT in the treatment population described here. Addition of chemotherapy to the adjuvant treatment protocol also is associated with an improved disease specific survival shown by the HR of 1.97 for S-RT compared to S-CRT.

The survival outcomes described herein definitively show a significant differences in survival outcomes between the different treatment groups. S-CRT offered the best survival outcome with 90% and 71% 2 and 5 year disease specific survival. These values represent improvements in disease specific survival of 16 and 33% compared to S-RT and CRT at 2 years, with improvements in survival of 17 and 22% compared to S-RT and CRT at 5 years. Cox regression analysis does confirm a statistically significant difference in hazard ratios between the different treatment groups indicating a significant survival benefit associated with S-CRT compared to S-RT, and CRT.

Information on patient co-morbidity and pre-treatment performance status was not fully available on all patients included in this analysis and therefore was excluded from the survival analysis. This was done to avoid any possible perceived selection bias and is tempered by the fact that this is a population study including all persons treated for advanced OPSCC in a single population and patients did self-select which multi-modality therapy they received following counseling in a multi-disciplinary treatment clinic.

Currently the HPV status of all patients examined during this study is unknown. Due to the findings that HPV positive OPSCC are associated with improved survival outcomes it is difficult to determine what this prognostic factors effect was on treatment related outcomes in the population described here [10-12]. A potential criticism of the survival outcomes presented here is that there could be a large number of HPV positive OPSCC represented in the S-CRT treatment group with the large survival benefit being possibly related to HPV status. However, a recent study completed at our center examining the HPV status and epigenetics of advanced OPSCC in all patients treated with S-CRT between 2006 and 2008 did show an HPV positivity rate of 48% in this patient population (S-CRT treatment group) [37] which is well under the estimated 72.2% prevalence of HPV positivity in advanced OPSCC from a recent meta-analysis of world literature [12,38]. If this 48% prevalence rate is extrapolated back to all patients within the S-CRT arm the high rates of 2 and 5 year overall and disease specific survival cannot be explained by HPV status, rather it is more likely due to the treatment related factors of combination of surgery followed by adjuvant therapies that account for the improved survival.

To build on the analysis presented within this manuscript we are currently undertaking HPV/p16 analysis on all patients examined here to more definitively examine the relationship of HPV status and treatment and survival outcomes. We are also examining the survival and demographics data of the second cohort of patients treated at the other tertiary care facility in the province to see if the relationships between treatment modality and survival outcome are reproduced at the other territorial treatment site.

## Conclusions

OPSCC remains a devastating disease presenting a treatment challenge to medical, radiation and surgical oncology treatment teams. In populations similar to the one described herein patients suffering from stage III or IV OPSCC patients enrolled in S-CRT treatment protocols did have the highest survival outcome compared to other treatment modalities. S-CRT is associated with a 17–22% 5 year disease specific survival benefit compared to CRT or S-RT.

This analysis of a longitudinal prospectively collected cancer registry does provide valuable information regarding the treatment of OPSCC and provides a potential useful benchmark for evidence-based counseling of patients suffering from OPSCC. It is imperative that all health professionals involved in the treatment of OPSCC acknowledge that any retrospective review of survival data cannot show causal relationships between survival outcomes and treatment modalities, rather only associations can be examined. As noted in the treatment population, patients with advanced stage OPSCC treated with S-CRT were associated with statistically significant increases in disease specific survival rates at both 2 and 5 years. The more challenging task arising from this survival analysis is how to utilize this data to predict future survival outcomes. With the lack of randomized control trials available one cannot definitively state that S-CRT offers an improved survival rate compared to S-RT or CRT, however the best available evidence on the treatment population described here does show an association between triple modality therapy (S-CRT) and improved rates of survival. The data presented here does support our current practice of offering patients with advanced stage OPSCC aggressive surgical resection and reconstruction followed by adjuvant chemoradiation therapy as a possible treatment option providing excellent chances of disease free survival and raises questions on what treatment modalities provide patients with advanced OPSCC with the optimum chances of disease free survival.

The survival outcomes presented here does generate multiple questions that require further investigation including what role does HPV status play in the relationship between survival outcome and treatment modality as well as is the association between increased disease specific survival and treatment with S-CRT compared to CRT noted in other treatment cohorts. Both of these questions are currently being examined by our research group with the hopes of providing more information regarding the optimum treatment of advanced OPSCC.

## Competing interests

The authors declare that they have no competing interests.

## Authors’ contributions

HS, JH, and RS provided patient data, mentorship, and editorial support of the manuscript. RM provided statistical analysis, data collection, and drafted the manuscript. CF, AK, and TM carried out data collection and manuscript editing. DO provided patient data, mentorship, secured ethics approval, drafted the manuscript and maintains data per local health ethics research office criteria. All authors have read and approved the final manuscript.

## Acknowledgments

All authors certify that the data and material presented within this manuscript has not been published in another peer reviewed journal and is not currently under consideration for publication in another journal.
